# Overcoming mental health challenges in higher education: a narrative review

**DOI:** 10.3389/fpsyg.2024.1466060

**Published:** 2024-12-11

**Authors:** Zamira Hyseni Duraku, Holly Davis, Aliriza Arënliu, Fitim Uka, Vigan Behluli

**Affiliations:** ^1^Department of Psychology, University of Prishtina, Prishtinë, Kosovë; ^2^University Counseling Service, The University of Iowa, Iowa City, IA, United States; ^3^Dots Counselling, Prishtinë, Kosovë

**Keywords:** mental health, higher education, mental health barriers, policy recommendations, student well-being, academic engagement, mental health services

## Abstract

**Background:**

Mental health among higher education students is a critical public health concern, with numerous studies documenting its impact on student well-being and academic performance. However, comprehensive research on the factors contributing to mental health deterioration, including barriers to seeking psychological help, remains insufficient. Gathering evidence on this topic is crucial to advancing policies, advocacy, and improving mental health services in higher education.

**Objective:**

This review explores the unique challenges faced by vulnerable student groups and highlights the factors influencing student well-being and academic engagement, including those exacerbated by the COVID-19 pandemic. The review also addresses barriers to accessing mental health services across various regions and provides evidence-informed recommendations for improving mental health policies and services in higher education, covering both well-researched and underexplored contexts.

**Methods:**

This narrative review synthesizes findings from over 50 studies on mental health in higher education. A targeted search was conducted using PubMed, Google Scholar, PsycINFO, CINAHL, and Scopus for studies published between 2013 and 2023. Data were analyzed through a deductive thematic content analysis approach, focusing on key predetermined themes related to student well-being, barriers to mental health services, and recommendations for policy improvements.

**Results:**

Several factors influence the mental health of higher education students, with vulnerable groups—including women, minorities, socioeconomically disadvantaged, international, and first-year students—experiencing higher levels of depression, anxiety, and stress. Factors that impact students’ well-being and academic performance include academic pressure, financial stress, lack of social support, isolation, trauma, lack of inclusive practices, and pandemic-related stressors. Institutional barriers, inconsistent well-being measures, data-sharing issues, and regulatory limitations hinder students’ access to mental health services, while stigma and lack of trust in mental health professionals impede care.

**Conclusion:**

Improving mental health strategies in higher education requires enhancing mental health services, addressing socioeconomic inequalities, improving digital literacy, standardizing services, involving youth in service design, and strengthening research and collaboration. Future research should prioritize detailed intervention reports, cost analyses, diverse data integration, and standardized indicators to improve research quality and applicability.

## 1 Introduction

Mental health among higher education students continues to be a significant global concern, with numerous studies documenting its impact on student well-being and academic performance. (Auerbach et al., 2016) reported that approximately 20% of college students worldwide develop mental health disorders within their first year of study, including major depression and anxiety disorders. Research across various regions supports this trend. The COVID-19 pandemic has further exacerbated mental health challenges, with notable issues reported in the United States (Lipson et al., 2023), Europe and the United Kingdom (Allen et al., 2022), and Eastern Europe, including Poland and Ukraine (Długosz et al., 2022; Rogowska et al., 2021). Studies from Southern Europe, including Kosovo, Albania, Serbia, and North Macedonia (Arënliu et al., 2021; Hyseni Duraku et al., 2023a; Mancevska et al., 2020; Pilika et al., 2022; Radovanovic et al., 2023), have also noted increased levels of anxiety, depression, and stress among university students. Alonso et al. (2018) also found that university students in multiple countries are experiencing a variety of mental health disorders, such as major depression, generalized anxiety, and panic disorder. Similar trends have been observed in South Africa (Bantjes et al., 2023).

The field of student mental health has evolved significantly over the past few decades, with early research primarily focusing on foundational aspects such as identifying basic stressors and psychological pressures inherent in higher education (e.g., academic workload, transitional stress). These studies, largely based in developed regions, laid the groundwork for understanding the unique mental health challenges students face and were instrumental in establishing university counseling services as the primary support system (Abelson et al., 2022). From the early 2000s onwards, research expanded to incorporate specific at-risk groups, such as first-year and minority students, whose mental health was found to be disproportionately affected due to factors like social isolation, financial strain, and lack of culturally sensitive resources (Stoll et al., 2022). This period also saw a shift toward understanding the institutional and systemic barriers that hinder access to mental health support, such as stigma, inconsistent policies, and varying service quality (Auerbach et al., 2018; Gaebel et al., 2021).

Despite continuous reports on the presence of mental health issues among higher education students across various countries, comprehensive research on the factors contributing to mental health deterioration, including barriers to seeking psychological help, remains limited. Furthermore, previous research has predominantly focused on developed countries, particularly the US, the European Union (EU), and the UK, leaving a gap in the literature concerning developing regions, including Southern Europe (Pilika et al., 2022; Radovanovic et al., 2023; Rogowska et al., 2021). By incorporating new studies from previously overlooked contexts, this contribution builds upon existing evidence and enhances the understanding of factors that need to be considered, as well as the importance of advancing mental health support in higher education. A more evidence-informed approach is essential, not only for improving mental health services but also for guiding the effective use of limited resources in policy development and advocacy (Abelson et al., 2022).

Between 2013 and 2023, the student mental health landscape has been further shaped by global events like the COVID-19 pandemic, which intensified pre-existing mental health challenges and introduced new stressors, including social isolation, remote learning demands, and uncertainty (Riboldi et al., 2023; Allen et al., 2022). Unlike earlier periods, recent studies have increasingly focused on identifying scalable, cost-effective solutions like digital interventions and peer support models to address these challenges across diverse socioeconomic and cultural contexts (Broglia et al., 2023; Özer et al., 2024). Furthermore, the field has witnessed a greater emphasis on systemic reforms, such as policy standardization, data-sharing improvements, and the inclusion of youth in the co-design of mental health services (Lynch et al., 2024; Bantjes et al., 2022). This analysis of historical and recent trends reveals both continuities and shifts, emphasizing the need for further research that not only addresses emerging mental health needs but also integrates the lessons learned from past interventions to enhance student support frameworks globally.

This review highlights the unique challenges vulnerable student groups face and explores the factors that influence student well-being and academic engagement, both in general and in specific periods, such as the recent COVID-19 pandemic. We also analyze barriers to accessing mental health services and provide evidence-based policy recommendations for improving mental health services in higher education. Policymakers, university administrators, mental health professionals, and academic staff can benefit from these insights and use them to develop more effective and inclusive mental health strategies and ensure better support for diverse and vulnerable student populations. These insights can also advance international collaboration to reform educational systems with the goal of protecting student well-being and reforming health systems to focus more on prevention, a proven and promoted approach to public health efficiency (World Health Organization, 2022).

## 2 Methods

The primary aim of this narrative review was to explore the factors influencing higher education students’ mental health, including mental health policies, services, and the suitability of higher education practices related to student well-being and mental health support. Given the diversity of the studies and regions covered—ranging from well-researched areas like the US, Europe, and the UK to underexplored regions such as South Africa and Southern Europe—this approach provided the flexibility to capture emerging insights and address significant gaps in the literature (Sukhera, 2022). Moreover, the narrative review method allowed for a thorough integration of the current state of knowledge, while adding new perspectives and offering a context-specific interpretation of the factors shaping mental health in higher education by consolidating various findings into a single review (Rumrill and Fitzgerald, 2001).

### 2.1 Inclusion and exclusion criteria

To maintain the rigor and focus of this narrative review, we established the following inclusion and exclusion criteria. We included English peer-reviewed studies, research reports, and gray literature (e.g., reports from reputable health organizations) that addressed factors influencing students’ mental health, mental health policies, services, and higher education practices. Reputable sources were defined as those from well-established global health organizations, such as the World Health Organization (WHO), and global mental health initiatives, which are widely recognized for their expertise in mental health policy and services. The studies covered a broad geographic area, including both developed and developing regions, and spanned a 10-year period (2013–2023), during which significant developments occurred in the field of mental health and higher education, particularly due to global events such as the COVID-19 pandemic. Studies that did not pertain to higher education or mental health services, non-peer-reviewed sources (excluding reputable health organizations and global mental health initiatives), opinion pieces, and publications outside the specified time frame were excluded.

### 2.2 Search strategy

This narrative review aimed to comprehensively synthesize global research on mental health in higher education. To identify relevant studies, we conducted a targeted search across multiple databases, including PubMed, Google Scholar, PsycINFO, CINAHL, and Scopus. PubMed was chosen for its robust collection of peer-reviewed health and mental health literature, particularly in psychology and psychiatry. At the same time, Google Scholar was selected to incorporate gray literature and regionally published studies, especially from developing regions. Expanding to additional databases—PsycINFO, CINAHL, and Scopus—allowed us to access a broader range of mental health and educational research pertinent to higher education.

To ensure both breadth and precision, the search incorporated terms and phrases aligned with the study’s objectives, such as “mental health in higher education,” “university counseling services,” “student mental health policies,” and “barriers to mental health services.” Boolean operators (“AND” and “OR”) were systematically used to combine terms and expand the search scope across each database. Related terms (e.g., “psychological support in universities,” “higher education mental health services”) and keywords specific to mental health services, policies, and vulnerable student groups were included to capture diverse terminology and interdisciplinary studies.

A detailed database search was performed, with special focus on inclusion and exclusion criteria and the number of articles retrieved, screened, and included. Titles and abstracts were screened for relevance, followed by a full-text review of selected studies. The final inclusion criteria focused on peer-reviewed studies, research reports and global project evaluations, published between 2013 and 2023 that addressed mental health, psychological support, policies, and barriers in higher education.

Though not a systematic review, this search strategy ensured comprehensiveness by balancing broad database selection with clearly defined inclusion criteria tailored to the review’s focus. This approach allowed for an in-depth examination of both well-researched and emerging contexts in student mental health across global higher education settings.

### 2.3 Data extraction and analysis

This review applied deductive thematic content analysis to ensure data were consistently aligned with the study’s predefined main objective. Following established guidelines for deductive thematic analysis (Braun and Clarke, 2006; Elo and Kyngäs, 2008), we focused on three primary themes identified in the preliminary literature review: (1) factors influencing student well-being and academic engagement, (2) barriers to accessing mental health services in higher education, and (3) evidence-based policy recommendations for improving mental health support in university settings.

During data extraction, the lead author examined each study’s objectives, methodologies, findings, and recommendations, identifying relevant data points that aligned with these themes. Data were then organized under specific subthemes such as “financial barriers,” “pandemic-related stressors,” and “support structures,” ensuring a nuanced understanding of the factors affecting student mental health. This approach enabled a structured analysis while allowing the flexibility to add inductively emerging subthemes that offered additional insights.

Throughout the coding process, we used a directed content analysis approach (Hsieh and Shannon, 2005), systematically mapping extracted data to the predefined themes and subthemes. Co-authors provided oversight to maintain accuracy and consistency in categorizing data. While primary themes were deductively applied, subthemes were included inductively if they contributed meaningful insights within the review’s scope. This structured yet flexible approach ensured a comprehensive synthesis of findings across studies and maintained methodological rigor in data extraction and analysis.

## 3 Results

This review differentiated over 50 studies to provide a comprehensive overview of mental health issues among higher education students and represent a wide range of regions, including the UK and other Western European countries, several Eastern European countries, the US, Australia, and South Africa. The studies focused on diverse populations within the context of higher education. Study populations included college and university students from different fields, years, and levels of studies: undergraduate, masters, and PhD students (including non-students in the same age range and those who had recently left college without graduating), and service providers for university students. Some of the studies focused on the global population, spanning multiple countries and regions, whereas others made comparisons with the general population.

The review encompassed a wide variety of study designs, including cross-sectional studies, qualitative studies, reviews, critical analyses, conceptual and intervention studies, randomized controlled trials (RCTs), system-level monitoring programs, case studies, systematic reviews, meta-analyses, brief reports, overviews, retrospective analyses, qualitative analyses, bibliometric mapping, parallel mixed-method designs, scoping reviews, and panel studies. We also included research reports conducted by leading health organizations and published project evaluation reports conducted by global mental health initiatives on monitoring the quality of mental health care in different countries.

The sample sizes of the reviewed studies varied significantly. Some studies involved extensive samples, such as a cross-sectional study with 192,202 students across 277 campuses, and others with sample sizes of 13,984 and 14,348 participants. The sample sizes of midrange studies were between 2,006 and 6,452 participants. Qualitative studies typically had smaller sample sizes such as 32 and 18 participants. Specific examples included a case study involving 27 professional staff members and an RCT with 1,200 participants. Table 1 summarizes the main characteristics of the studies.

**Table 1:** Summary characteristics of included studies.

Study	Country	Population	Study design	Sample size
Abelson et al. (2022)	United States	University students	Multidisciplinary review	N/A
Allen et al. (2022)	UK, Italy, Germany and Spain	University students	Cross-sectional	2,006
Alonso et al. (2018)	Multiple countries	University Students (first year)	Cross-sectional	13,984
Arday (2018)	UK	Black and ethnic minority university students	Qualitative study	32
Arënliu et al. (2021)	Kosovo	University students	Cross-sectional	904
Auerbach et al. (2016)	21 countries	College students, non-students in the same age range (18–22 years), non-students who recently left college without graduating	Cross-sectional	6,452
Auerbach et al. (2018)	Australia, Belgium, Germany, Mexico, Northern Ireland, South Africa, Spain, and US	University students	Cross-sectional	13,984
Auerbach et al. (2019)	Australia, Belgium, Germany, Mexico, Northern Ireland, South Africa, Spain, and the US	First-year college students	Cross-sectional	14,348
Banks (2020)	United States	University students	Cross-sectional	265
Bantjes et al. (2022)	Global	University students	Review	N/A
Bantjes et al. (2023)	South Africa	University students	Critical analysis	N/A
Barrable et al. (2018)	Greece	University students	Intervention study	N/A
Berman et al. (2024)	Sweden	University students	RCT	1,200
Bíró et al. (2019)	Hungary	Higher education students	Cross-sectional	409
Bramesfeld et al. (2016)	Eight EU Countries	Quality monitoring programs in mental health care	System-level monitoring	N/A
Broglia et al. (2023)	UK	Service providers of university students	Case study	27 professional staff
Bruffaerts et al. (2018)	Belgium	College Freshmen	Cross-sectional	4,921
Campbell et al. (2022)	UK	University and college students	Systematic review	N/A
Clarke et al. (2015)	Various	Youth (12–25 years old)	Systematic review	25 studies
Conley et al. (2016)	Various	University students	Meta-analysis	48 studies
Conley et al. (2017)	Multiple countries	At-risk higher education students	Meta-analysis	N/A
Cuijpers et al. (2019a)	Multiple countries	College students	Brief report	N/A
Cuijpers et al. (2019b)	Multiple countries	College students	Overview	N/A
Cuijpers et al. (2023)	Multiple countries	Global population	Report	N/A
Davies et al. (2014)	Multiple countries	University students	Systematic review and meta-analysis	17 RCTs
De Lima et al. (2021)	Countries with initial high income, low- and middle-income countries	Mental health services	N/A	N/A
Defeyter et al. (2021)	UK	University students	Cross-sectional	600
Długosz et al. (2022)	Poland and Ukraine	Young students	Cross-sectional	2,022
Dodd et al. (2021)	UK	University students	Scoping review	N/A
Dougall et al. (2023)	UK	University students	Cross-sectional	811
Ebert et al. (2019a,b)	Germany	University Students	RCT	1,374
Ebert et al. (2019a,b)	Australia, Belgium, Germany, Mexico, Northern Ireland, South Africa, Spain, and the US	First-year college students	Cross-sectional	13,984
Farrer et al. (2013)	Multiple countries	University students	Systematic review	27 RCTs
Franzoi et al. (2022)	Europe	University students	Retrospective analysis	N/A
Gaebel et al. (2021)	Belgium, France, Germany, Ireland, the Netherlands, and the UK	eMEN project^a^	Qualitative analysis	N/A
Guzman Villegas-Frei et al. (2024)	Switzerland	University students	Cross-sectional	2,415 first- and second-year undergraduate students
Harrer et al. (2019)	Multiple countries	University students	Systematic review and meta analysis	48 RCTs
Hernández-Torrano et al. (2020)	Multiple countries	University students	Bibliometric mapping	Various
Hyseni Duraku et al. (2023a)	Kosovo	University students	Parallel mixed-method design	234
Hyseni Duraku et al. (2023b)	Kosovo	University students and parents	Cross-sectional	121 parents, 116 students
Kovess-Masfety et al. (2016)	France	College students and non-students	Cross-sectional	2,424
Levecque et al. (2017)	Belgium	PhD students	Cross-sectional	3,659
Lipson et al. (2023)	US	University students	Cross-sectional	192,202 students from 277 campuses
Lynch et al. (2024)	Ireland	Young people	Qualitative study	18
Mancevska et al. (2020)	North Macedonia	University students	Cross-sectional	280
Matos Fialho et al. (2021)	Germany	University students	Cross-sectional	5,021
Nurunnabi et al. (2020)	G20 countries (19 countries and EU)	University students	Review article	N/A
Oliveira et al. (2021)	Multiple countries	University students	Systematic review	N/A
Özer et al. (2024)	Turkey	University students	Cross-sectional	273
Pilika et al. (2022)	Albania	University students	Cross-sectional	570
Quimby and Agonafer (2023)	United States	University students	Conceptual/theoretical study	N/A
Radovanovic et al. (2023)	Serbia	University students	Cross-sectional	588
Riboldi et al. (2023)	Italy and UK	University students	Qualitative comparative study	32
Riboldi et al. (2024)	Italy and UK	University students	Qualitative	33
Rogowska et al. (2021)	Poland	University students	Cross-sectional	1,961
Slimmen et al. (2022)	Netherlands	University students	Cross-sectional	875
Stoll et al. (2022)	UK	Black university students	Qualitative thematic synthesis of a literature review	Various
Tabor et al. (2021)	UK	University students and non-students	Panel study	11,519
Thom et al. (2021)	Germany	General population	Framework development	N/A
World Health Organization (2022)	Global	Global population	Report	N/A

a Project introduction was initiated as a six-country project in 2016, funded by the European Regional Development Fund within the funding area Interreg North-West Europe The project aimed to increase the dissemination and quality of eMH services. As part of the project’s activities, a transnational policy was formulated with recommendations for upscaling eMH throughout the EU and beyond. The transnational policy maps out barriers and facilitators for the implementation of eMH, and proposes actions for EU policymakers and other eMH.

### 3.1 Theme 1: factors influencing students’ well-being and academic engagement

The subthemes summarize various factors that significantly impact the mental health and academic engagement of higher education students, as identified in the reviewed studies. These factors include mental health issues, barriers to seeking professional help, academic pressure, social support and isolation, financial difficulties, specific cultural and behavioral influences, pandemic-related stressors, and issues faced by minority and vulnerable groups, including intersectionality.

#### 3.1.1 Consequences of mental health issues and barriers to seeking professional help

Auerbach et al. (2016) found that approximately 20% of college students globally had been diagnosed with mental disorders lasting 12 months or more, with the majority having an onset before entering college; these diagnoses were strongly associated with college dropouts. Berman et al. (2024) concluded that mood and anxiety disorders are key predictors for higher education dropout, with a significant treatment gap in which many students do not seek help. Hyseni Duraku et al. (2023a) found perceived social support and academic anxiety were significant predictors of Kosovar students’ barriers to seeking psychological help.

Studies from Hungary, Kosovo, Albania, North Macedonia, Poland, and Serbia showed that female students experience higher levels of depression, anxiety, and stress than their male counterparts. The COVID-19 pandemic exacerbated these issues, particularly among female students, due to increased stressors and challenges and a lack of psycho-emotional support from their universities (Arënliu et al., 2021; Bíró et al., 2019; Mancevska et al., 2020; Pilika et al., 2022; Radovanovic et al., 2023; Rogowska et al., 2021).

Freshmen and international students also face significant mental health challenges, particularly during the COVID-19 pandemic period. Isolation, loneliness, and poor communication with universities exacerbate these issues, making students particularly vulnerable to mental disorders. Several studies across multiple countries have shown a high prevalence of mental disorders among first-year students (Auerbach et al., 2016; Bruffaerts et al., 2018; Riboldi et al., 2023).

#### 3.1.2 Influence of academic pressure and changes in learning systems

Bruffaerts et al. (2018) noted that academic pressure and the transition to university negatively affected the mental health of freshmen in Belgium. Levecque et al. (2017) highlighted high workloads and academic pressure as significant stressors among PhD students in Belgium. Slimmen et al. (2022) found that perceived stress drastically reduced mental well-being among university students in the Netherlands, with academic pressure having the greatest negative impact, followed by stress related to extra-curricular activities and financing. High-risk groups, such as students with multiple mental disorders, experience severe role impairment, and are strongly associated with college dropout rates (Alonso et al., 2018).

The high demands of academic work and the need to adapt to a new environment have significantly contributed to stress levels among students in the US (Abelson et al., 2022). Similarly, Matos Fialho et al. (2021) reported a notable increase in stress among German university students due to their academic workload and concerns about completing their studies during the COVID-19 pandemic. The shift to online learning and changes in teaching methods further intensified this pressure. In Serbia, Radovanovic et al. (2023) also identified significant levels of depression, anxiety, and stress among university students, also linked to the impact of COVID-19. The abrupt transition to remote learning environments and the uncertainty surrounding the pandemic contributed to elevated stress levels.

#### 3.1.3 Influence of social support and isolation during the COVID-19 pandemic

Nurunnabi et al. (2020) and Bruffaerts et al. (2018) showed that social distancing and self-isolation during the COVID-19 pandemic exacerbated stress among students. The lack of face-to-face interaction and support networks heightened feelings of loneliness and anxiety. Nurunnabi et al. (2020), Allen et al. (2022), and Arënliu et al. (2021) found that lockdown was one of the key contributors to mental health challenges among students during COVID-19. Riboldi et al. (2023) highlighted that generalized social anxiety among university students in Italy and the UK during the COVID-19 pandemic was linked to loneliness, excessive online time, unhealthy management of time and space, and poor communication with learning institutions. Vulnerable groups included freshmen, international students, and students at the extremes of the introversion/extroversion spectrum.

#### 3.1.4 Financial barriers

Abelson et al. (2022) reported that financial stress, exacerbated by student debt and concerns about job uncertainty, is a major contributor to mental health struggles among students in the US. Similarly, Lipson et al. (2023) found that although first-generation students in the US have higher levels of depression and anxiety, they use mental health services significantly less than continuing-generation students. Barriers such as financial constraints and a lack of awareness of available services contribute to this disparity. Socioeconomic inequalities in the UK and financial difficulties in Poland, Ukraine, and Kosovo were also shown to negatively affect students’ mental health (Arënliu et al., 2021; Długosz et al., 2022; Dougall et al., 2023; Lipson et al., 2023).

#### 3.1.5 Lack of culturally competent support, discrimination, and marginalization

Students often face additional stress due to discrimination and social isolation. In the US, Abelson et al. (2022) reported that racial and gender-based discrimination are strong risk factors, particularly for students of color and LGBTQ+ students, contributing significantly to psychological distress and anxiety. Similarly, in the UK, Stoll et al. (2022) demonstrated that university students of color face exacerbated mental health challenges due to academic pressure, racism, and a lack of culturally competent support. Arday (2018) further highlighted significant barriers to accessing mental health services among students of color and ethnic minority students in the UK, including a lack of cultural understanding and communication, which negatively impacts their mental health and academic outcomes. Sexual minorities and transgender students, across multiple countries, encounter similar mental health challenges due to family rejection, bullying, and social isolation. Despite their greater need for mental health services, these students are often less likely to utilize available resources. Mental health disorder comorbidity within this group is strongly associated with increased suicidal thoughts and behaviors. These findings are consistent across studies conducted in Australia, Belgium, Germany, Mexico, Northern Ireland, South Africa, Spain, and the US (Auerbach et al., 2016).

#### 3.1.6 Cultural, behavioral, and trauma related factors affecting student mental health

Separation from the family unit, especially for students from cultures with strong family ties, increases the risk of mental health issues. Culture shock, particularly among first-generation students, further complicates their adjustment and contributes to mental health struggles (Kish, 2003, as cited in Abelson et al., 2022). Experiences of trauma and assault are also significant risk factors. Students who have experienced assault are at much higher risk of developing conditions such as PTSD, anxiety, and depression (Khadr et al., 2019; Lilly et al., 2011, as cited in Abelson et al., 2022). Additionally, research shows that students whose parents had perceived PTSD are more prone to experiencing PTSD symptoms and report experiencing more traumatic situations, including sexual assault (Hyseni Duraku et al., 2023b). Behavioral factors, such as substance use, including binge drinking and marijuana use, are commonly associated with mental health problems. Unhealthy sleep habits are both a cause and a consequence of mental health issues, significantly impacting students’ well-being and academic performance (Morin et al., 2011, as cited in Abelson et al., 2022). Table 2 summarizes the factors that influence students’ well-being and academic engagement.

**Table 2:** Factors that influence students’ well-being and academic engagement.

Sub-theme	Key findings	Countries and regions	Most vulnerable groups	Sources
Consequences of Mental Health Issues and Barriers to Seeking Professional Help	High prevalence of mental disorders, predictors of college dropout Treatment gap with many students not seeking help Academic anxiety and perceived social support are key predictors for seeking help	Global, Kosovo, Hungary, Albania, North Macedonia, Poland, Serbia, Australia, Belgium, Germany, Mexico, Northern Ireland, South Africa, Spain, and US	Female students, freshmen, international students	Auerbach et al. (2018), Bruffaerts et al. (2018), Bíró et al. (2019), Mancevska et al. (2020), Arënliu et al. (2021), Rogowska et al. (2021), Pilika et al. (2022), Hyseni Duraku et al. (2023a), Radovanovic et al. (2023), Riboldi et al. (2023), Berman et al. (2024)
Influence of academic pressure and changes in learning systems during the COVID-19 pandemic	Academic pressure, high workload, transition to university life, online learning challenges, higher stress levels	US, Belgium, Germany, Netherlands, Serbia	PhD students, general university students, students with multiple mental disorders	Levecque et al. (2017), Alonso et al. (2018), Bruffaerts et al. (2018), Matos Fialho et al. (2021), Slimmen et al. (2022), Radovanovic et al. (2023), Abelson et al. (2022)
Influence of social support and isolation during the COVID-19 pandemic	Social distancing, lack of face-to-face interaction, loneliness, anxiety, limited support, lockdowns	Poland, Ukraine, Italy, UK, Kosovo	Freshmen, international students, students at the extremes of the introversion/extroversion spectrum	Bruffaerts et al. (2018), Nurunnabi et al. (2020), Długosz et al. (2022), Riboldi et al. (2023), Arënliu et al., 2021
Financial barriers	financial issues, concerns about students’ debts, job-insecurity, lower use of services, higher mental health struggles, higher levels of depression and anxiety	US, UK, Poland, Ukraine, Kosovo	First-generation students, students from low socioeconomic backgrounds	Abelson et al. (2022), Nurunnabi et al. (2020), Arënliu et al. (2021), Allen et al. (2022), Długosz et al. (2022), Dougall et al. (2023), Lipson et al. (2023)
Lack of culturally competent support, discrimination, marginalization	Racism, lack of cultural support, discrimination, communication issues, mental health challenges, suicidal thoughts	UK, Australia, Belgium, Germany, Mexico, Northern Ireland, South Africa, Spain, US	Black and ethnic minority students, sexual minority, transgender students	Abelson et al. (2022), Arday (2018), Auerbach et al. (2018), Stoll et al. (2022)
Cultural, Behavioral, and Trauma Related Factors Affecting Student Mental Health	Separation from family, culture shock, trauma, assault, Intergenerational trauma substance use, binge drinking, substance use, unhealthy sleep habits	US, Canada, UK, Kosovo	First generation students, first year students, undergraduate students	Kish (2003), as cited in Abelson et al. (2022), Khadr et al. (2019), Lilly et al. (2011), as cited Abelson et al. (2022), Hyseni Duraku et al. (2023b), Morin et al. (2011), ac cited in Abelson et al. (2022)

### 3.2 Theme 2: barriers to quality mental health services for university students and young adults

This section describes the main barriers affecting access to mental health services for university students and young adults, including national policy gaps, regulatory limitations, and social and cultural challenges.

#### 3.2.1 National policies, regulations, and availability of services

Globally, there are significant gaps in the availability of mental health services due to a shortage of personnel and services that are often unaffordable. High-income countries face inefficiencies in mental health services, whereas low- and middle-income countries suffer from poor access and inadequate resources (de Lima et al., 2021). Regulatory frameworks are often limited and provide inadequate support for students and young adults during critical transitions and lead to fragmented federal policies (Gaebel et al., 2021; Kovess-Masfety et al., 2016). Effective management and policy improvements depend on mental health indicators; however, their effectiveness remains uncertain, with many initiatives still in the pilot stage (de Lima et al., 2021). Structural barriers, such as cost and logistical issues, significantly influence the likelihood of individuals seeking psychological help (Ebert et al., 2019a,b).

#### 3.2.2 Higher education institutions’ well-being support, the quality of services, and inclusive practices

There is a lack of standardization in defining and measuring student well-being. Institutions often use non-standardized and single-item indicators that do not adequately include university-level indicators (Dodd et al., 2021). Restrictions on information-sharing and an incompatible data infrastructure create barriers to meeting students’ mental health demands (Broglia et al., 2023). Many institutions in European countries do not provide adequate mental health counseling services. Geographical disparities also exist, and medium-sized institutions are more likely to have University Counseling Centers (Franzoi et al., 2022; Hyseni Duraku et al., 2023a; Mancevska et al., 2020; Pilika et al., 2022; Radovanovic et al., 2023). In the US, while more mental health services are available, higher education institutions often have higher demands compare to their resources, which leads to longer wait times and poorer outcomes for students (Abelson et al., 2022). Furthermore, in many countries mental health services available often fail to meet the needs of vulnerable groups such as ethnic minorities, sexual minorities, first-generation students, and freshmen (Auerbach et al., 2018; Banks, 2020; Lipson et al., 2023; Lynch et al., 2024; Stoll et al., 2022).

#### 3.2.3 Social and cultural barriers

A lack of cultural understanding and communication issues negatively impact the mental health and academic outcomes of students. In many countries, especially in Southern Europe and the UK, stigma and a lack of trust lead to hesitation in self-disclosure and seeking help (Hyseni Duraku et al., 2023a). In the US, although personal stigma around mental health has decreased, it still influences whether students choose to seek help (Abelson et al., 2022).

#### 3.2.4 Technological and awareness barriers

Although digital interventions have potential, most students are unaware of Internet-based interventions and prefer human interaction. Challenges exist in the efficacy, personalization, and engagement of digital tools (Özer et al., 2024; Riboldi et al., 2024). Table 3 summarizes the themes and subthemes that represent barriers.

**Table 3:** Barriers to quality mental health services for university students and young adults.

Sub-code	Finding	Countries	Description	Sources
National policies, regulations, availability of services	Global gaps in mental health services	Global, low-income countries, high-income countries	Gaps in the availability of mental health services Unaffordable services Inefficient mental health services in high-income countries Poor access to mental health services in low- and middle-income countries	de Lima et al. (2021)
	Regulatory and policy limitations	Global	Limited legal and regulatory framework Fragmented federal policies	Kovess-Masfety et al. (2016), Gaebel et al. (2021)
	Lack of mental health indicators	Global	Many initiatives are in partial or pilot stages	de Lima et al. (2021)
	Cost and logistical issues	Global	Cost and logistical issues’ influence on the likelihood of students seeking help	Ebert et al. (2019a,b)
Higher education institutions well-being support, the quality of services, inclusive practices	Inconsistencies in well-being measurements	Global	Lack of standardization in the definition and measurement of student well-being Use of non-standardized measures and single-item indicators	Dodd et al. (2021)
	Data-sharing and infrastructure issues	Global	Restrictions on information sharing Incompatible data infrastructures	Broglia et al. (2023)
	Lack of university professional counseling services	EU & Southern Europe	Most institutions do not provide adequate mental health counseling services in EU countries and Southern Europe Higher probability of psychological centers in medium-sized institutions	Mancevska et al. (2020), Franzoi et al. (2022), Pilika et al. (2022), Hyseni Duraku et al. (2023a), Radovanovic et al. (2023)
	Lack of resources in university counseling services	US	Higher demand than available resources Longer wait times and poorer outcomes	Abelson et al. (2022)
	Lack of tailored support and inclusive practices	Global	Lack of tailored and inclusive practices, especially for vulnerable groups (ethnic minorities, sexual minorities, first-generation students, freshmen)	Auerbach et al. (2018), Stoll et al. (2022), Lipson et al. (2023), Lynch et al. (2024)
Social and cultural barriers	Stigma, cultural understanding, trust issues	US, UK, Kosovo	Despite a decrease in personal stigma around mental health, it continues to influence whether students in the US seek help Lack of cultural understanding and communication issues for minorities Stigma, lack of trust, self-disclosure hesitation among Kosovar students	Abelson et al. (2022), Arday (2018), Hyseni Duraku et al. (2023a)
Technological and awareness barriers	Digital interventions and awareness	Global	Lack of awareness of Internet-based interventions Preference for human interaction in interventions Challenges in efficacy, personalization, engagement of digital tools	Özer et al. (2024), Riboldi et al. (2024)

### 3.3 Theme 3: recommendations for enhancing mental health services for university students and young adults

The following narrative summarizes the key recommendations and highlights of the studies reviewed.

#### 3.3.1 Systemic improvements

De Lima et al. (2021) emphasized the importance of improving data systems, enacting policies and legislative reforms, reallocating resources, enhancing patient-centered care, and building capacity through training and coordination. Bramesfeld et al. (2016) highlighted the need for the systematic monitoring of mental health services across the EU to address disparities and ensure high-quality care. Thom et al. (2021) recommended a comprehensive framework and indicator set for mental health surveillance to improve data collection and standardize indicators in Germany. Dodd et al. (2021) called for standardized measures and a unified conceptual framework to accurately capture student well-being, emphasizing the development of a core set of well-being measures validated for students. Alonso et al. (2018) and Auerbach et al. (2016) stressed the importance of preventative interventions that target mental disorders and associated impairments and recommended their integration into broader public health strategies. Bantjes et al. (2022) advocated the conceptualization of public health interventions within a developmental paradigm that recognizes the unique developmental tasks of young adulthood and suggested novel, evidence-based approaches to scale-up services and adapt interventions to student-specific contexts.

#### 3.3.2 Strengthening research and collaboration

Hernández-Torrano et al. (2020) suggested initiatives, such as the World Mental Health International College Student Initiative, to strengthen national and international research partnerships and facilitate knowledge exchange. Broglia et al. (2023) recommended stronger partnerships between universities and the national health system (NHS) in the UK. Conley et al. (2017) advocated improving future research on mental health prevention programs by expanding the range of outcomes assessed and clarifying the moderators and mediators of intervention impact to benefit at-risk students in higher education. Abelson et al. (2022) highlighted the importance of continuing to collect and disseminate data to understand mental health needs in college populations, with a particular focus on identifying and addressing inequalities exacerbated by the pandemic, economic stress, and racism.

#### 3.3.3 Tailored interventions and participatory approaches

Matos Fialho et al. (2021) and Arënliu et al. (2021) recommended strategies to support students’ well-being during crises such as the COVID-19 pandemic. Stoll et al. (2022) and Arday (2018) emphasized the creation of culturally sensitive mental health programs and enhanced cultural competence training for healthcare providers. Auerbach et al. (2018) highlighted the need for inclusive support for transgender and sexual minority students. Ebert et al. (2019a,b) recommended personalized interventions tailored to individual barriers and clinical characteristics to increase university students’ intention to use mental health services. Lynch et al. (2024) emphasized the importance of youth participation in co-designing mental health services to ensure services are developmentally appropriate, relevant, and respectful. Quimby and Agonafer (2023) proposed a culturally responsive model for embedded counseling, advocating for mental health support within campus cultural centers to better serve the unique needs of marginalized student populations.

#### 3.3.4 Enhance digital literacy, peer to peer support, and digital mental health interventions

Nurunnabi et al. (2020) advocated the implementation of flexible online mental health support programs and timely interventions by health providers in G20 countries. Digital interventions and peer-to-peer approaches can present cost-effective ways to expand the range of available services, thereby increasing accessibility and providing support tailored to students’ specific needs (Bantjes et al., 2022). Transdiagnostic and tailored internet interventions are supported for reducing the treatment gap and enhancing academic performance (Berman et al., 2024; Davies et al., 2014; Harrer et al., 2019), while mobile app-based interventions are recommended to address student mental health needs and alleviate challenges related to limited human resources (Oliveira et al., 2021).

Cuijpers et al. (2019a,b, 2023) highlighted the importance of digital tools and interventions for mental health among students, and recommended the development of evidence-based digital tools, integrated community-based mental health services, and investment in task-sharing interventions. Özer et al. (2024) emphasized enhancing digital literacy and creating digital interventions that consider student preferences. Riboldi et al. (2024) advocated the integration of digital tools with face-to-face interventions using a multimodal approach. Gaebel et al. (2021) provided recommendations for the implementation of e-mental health services across Europe, stressing the need for political commitment, legal clarity, and increased digital health literacy. Clarke et al. (2015) demonstrated that skills-based interventions in a module-based format can significantly improve mental health outcomes for youth, particularly through the use of computerized cognitive behavioral therapy, which has shown positive effects on anxiety and depression. Similarly, Conley et al. (2016) and Farrer et al. (2013) emphasized the effectiveness of technological mental health prevention programs for students, showing that skill-training interventions are particularly beneficial for improving mental health in both general and at-risk student populations.

#### 3.3.5 Addressing socioeconomic inequalities and enhancing personal well-being

Dougall et al. (2023) urged academic institutions to develop policies to address socioeconomic inequalities in mental health and well-being. Guzman Villegas-Frei et al. (2024) suggested interventions to reinforce self-efficacy, mindfulness, and social support. Table 4 presents a detailed breakdown of these findings, including specific descriptions and sources.

**Table 4:** Recommendations for enhancing mental health services and well-being.

Category	Recommendations	Specific actions and examples
Systemic improvements	Improve data systems, policy and legislative reforms, resource allocation, patient-centered care, coordination, capacity building	Systematic monitoring of mental health services across EU countries (Bramesfeld et al., 2016); comprehensive framework and indicator set for mental health surveillance in Germany (Thom et al., 2021); standardized measures and unified conceptual framework for student well-being (Dodd et al., 2021); preventative interventions targeting mental disorders and associated impairments (Auerbach et al., 2016; Alonso et al., 2018; Conley et al., 2016); integrate preventative interventions in broader public health strategies (Auerbach et al., 2016; Alonso et al., 2018); conceptualize public health interventions within a developmental paradigm (Bantjes et al., 2022)
Strengthen research and collaboration	Continue data collection and address inequalities caused by the pandemic, economic stress and racism Strengthen national and international research partnerships and facilitate knowledge exchange	Importance of identifying and addressing inequalities in college populations (Abelson et al., 2022) Stronger partnerships between universities and national health services (Broglia et al., 2023); expand the range of outcomes assessed and clarify moderators/mediators of intervention impact (Conley et al., 2017)
Tailored interventions and participatory approaches	Support students’ well-being during crises like the COVID-19 pandemic, create culturally sensitive mental health programs, enhance cultural competence training for healthcare providers	Inclusive support for transgender and sexual minority students (Auerbach et al., 2018); personalized interventions tailored to individual barriers and clinical characteristics (Ebert et al., 2019a,b); youth participation in co-designing mental health services (Lynch et al., 2024); support during crises (Matos Fialho et al., 2021); culturally sensitive programs (Arday, 2018; Stoll et al., 2022); embedded counseling model within campus cultural centers (Quimby and Agonafer, 2023)
Enhance digital literacy, peer to peer support and digital interventions	Develop evidence-based digital tools, integrate community-based mental health services, enhance peer to peer approaches, invest in task-sharing interventions; enhance digital literacy and create digital interventions that consider student preferences	Flexible online mental health support programs and timely interventions by health providers in G20 countries (Nurunnabi et al., 2020); digital interventions and peer-to-peer approaches to expand available services (Bantjes et al., 2022); transdiagnostic and tailored internet interventions to reduce the treatment gap (Berman et al., 2024; Davies et al., 2014; Harrer et al., 2019); implement mobile app-based interventions (Oliveira et al., 2021), integrate digital tools with face-to-face interventions through a multimodal approach (Riboldi et al., 2024); implement e-mental health services across Europe (Gaebel et al., 2021); political commitment, legal clarity, and increased digital health literacy for e-mental health services (Gaebel et al., 2021) skills-based interventions in a module-based format, particularly computerized cognitive behavioral therapy, can significantly improve mental health outcomes for adolescents, showing positive effects on anxiety and depression (Clarke et al., 2015); skill-training technological interventions are beneficial for improving mental health in both general and at-risk student populations (Conley et al., 2016; Farrer et al., 2013)
Address socioeconomic inequalities and enhance personal well-being	Develop policies that address socioeconomic inequalities in mental health and well-being	Interventions to reinforce self-efficacy, mindfulness, and social support (Guzman Villegas-Frei et al., 2024)

## 4 Discussion

The reviewed studies consistently indicate that mental health challenges, such as anxiety, stress, depression, and academic-related anxiety, are widespread among university students. These issues significantly affect student well-being, contributing to increased dropout rates, especially among those experiencing multiple mental health disorders (Alonso et al., 2018; Auerbach et al., 2016; Berman et al., 2024; Hyseni Duraku et al., 2023a). The onset of the COVID-19 pandemic further exacerbated these problems, with vulnerable groups such as female students, freshmen, and international students experiencing heightened levels of distress (Arënliu et al., 2021; Bíró et al., 2019; Riboldi et al., 2023).

Several pervasive factors contribute to poor mental health across different regions. Common stressors include academic pressure, financial difficulties, lack of social support, substance use, family separation, and unhealthy sleep habits (Bruffaerts et al., 2018; Nurunnabi et al., 2020; Kish, 2003; Morin et al., 2011, as cited in Abelson et al., 2022; Hyseni Duraku et al., 2023b). Social support is identified as a crucial protective factor; students with adequate support are more likely to seek psychological help, while those lacking such support experience more severe mental health issues (Bruffaerts et al., 2018; Nurunnabi et al., 2020). Additionally, the lack of culturally sensitive services and persistent issues of discrimination further exacerbate these barriers, particularly for ethnic and sexual minority students, making them less likely to seek help (Stoll et al., 2022; Abelson et al., 2022).

Despite a number of globally recognized factors impacting student mental health, the studies reviewed also highlight differences in these factors across countries and among specific groups. For example, in both developed and developing countries, financial stress is frequently reported as a major factor affecting student well-being. However, in the United States, financial issues are predominantly driven by the high burden of student debt (Abelson et al., 2022; Lipson et al., 2023). Moreover, first-generation students in the US, despite facing significant financial hardships that negatively impact their mental health, also tend to use mental health services less frequently compared to continuing-generation students (Lipson et al., 2023). In contrast, in the European context, including the UK, financial difficulties are more closely tied to socioeconomic inequalities (Długosz et al., 2022; Dougall et al., 2023). In Southern Europe, financial challenges are further compounded by broader economic instability, which not only exacerbates mental health issues but also limits students’ access to mental health services (Arënliu et al., 2021). High service costs and limited financial aid options make it difficult for students to seek professional help, worsening their conditions (Arënliu et al., 2021; Hyseni Duraku et al., 2023b).

In Southern Europe, cultural stigmas surrounding mental health are more pronounced, and family-oriented values often increase reluctance to discuss mental health issues openly, further discouraging help-seeking behaviors (Hyseni Duraku et al., 2023a). In contrast, in the U.S. and the UK, discrimination based on race, gender, and sexual orientation serves as a significant barrier to accessing mental health services. Students of color and LGBTQ+ students face unique challenges related to marginalization and a lack of culturally competent support (Abelson et al., 2022; Stoll et al., 2022). In Turkey, limited awareness about digital mental health solutions reduces their potential effectiveness, highlighting the need for improved digital literacy and engagement (Özer et al., 2024). These regional variations underscore the importance of tailoring mental health interventions to address specific socioeconomic and cultural conditions unique to each context (Quimby and Agonafer, 2023). Furthermore, as recommended by the reviewed studies, involving students in the design and implementation of mental health programs is essential to ensure that these services are relevant and meet the actual needs of the student population (Lynch et al., 2024).

The findings also reveal several barriers to accessing quality mental health services for university students worldwide, yet these barriers differ depending on the context. While common obstacles include shortages of mental health personnel, high service costs, and fragmented healthcare policies, these issues are particularly problematic in low- and middle-income countries (de Lima et al., 2021; Kovess-Masfety et al., 2016). Additionally, higher education institutions face challenges such as inconsistent well-being assessments and a lack of standardized data-sharing systems, which hinder effective mental health support (Abelson et al., 2022; Dodd et al., 2021). However, such services are more widely available in the U.S. compared to many European countries, where access remains limited (Hyseni Duraku et al., 2023a; Hyseni Duraku et al., 2023b).

Similarly, methodological issues observed in the reviewed studies can impact the generalizability of findings and the identification of influential factors and prevalence rates among young adults and higher education students. The reviewed studies utilize diverse methodological approaches, including randomized controlled trials, cross-sectional studies, and qualitative research, each with distinct strengths and limitations. For instance, randomized controlled trials, like those conducted by Berman et al. (2024), provide robust evidence on intervention efficacy. However, cross-sectional studies that rely on self-reported data may introduce biases, especially in assessing symptoms such as anxiety and depression (Ebert et al., 2019a,b). Additionally, inconsistencies in the definitions and measurements of mental health outcomes, such as prevalence rates, complicate comparisons across studies (Bruffaerts et al., 2018). Moreover, the geographic focus of most studies is concentrated in high-income regions like the U.S., Europe, and Australia, leaving emerging regions such as Southern Europe and South Asia underrepresented (de Lima et al., 2021). This lack of data from diverse geographical contexts limits the global generalizability of the findings and highlights the need for more inclusive research to better capture the mental health needs of university students worldwide. Therefore, standardizing well-being measures across educational institutions is recommended to ensure consistent and comparable data, which are crucial for effective policy-making (Bramesfeld et al., 2016; de Lima et al., 2021). Furthermore, beyond addressing methodological issues, preventive measures integrated into broader public health strategies can facilitate early identification and intervention, particularly among young adults at critical stages of mental health development (Alonso et al., 2018). Strengthening international collaboration is also vital for bridging service gaps, improving data collection, and sharing best practices globally, thereby contributing to more comprehensive mental health strategies (Abelson et al., 2022; Hernández-Torrano et al., 2020).

### 4.1 Limitations and future direction

The findings of this study, which encompass a diverse range of studies from various contexts, provide valuable insights into promoting the importance of identification of influential factors on student mental health and the importance of their support. However, future research should adopt more systematic protocols and rigorous methodologies to improve the comparability of findings, which would, in turn, strengthen the evidence base needed to inform policy and service improvements within higher education institutions.

Furthermore, to capture a broader spectrum of student experiences and support service availability, future reviews should focus on analyzing both the policies and legal frameworks surrounding student and young adult mental health, as well as university mental health strategies from different global regions. Such an approach is critical not only for improving global representation but also for understanding how various institutional, legal, and cultural frameworks shape student mental health outcomes. Additionally, incorporating studies from non-English publications would provide valuable insights from underrepresented regions, thereby expanding the knowledge of mental health practices and barriers to care.

Another key consideration for future research is the variability in how mental health is understood across cultural contexts. Cross-cultural validation of terms and measures is essential to ensure consistency in interpreting mental health concepts across diverse populations, which would lead to more reliable and globally relevant findings. Additionally, distinguishing between general mental well-being and clinically diagnosed mental illnesses is crucial for designing more targeted interventions and better understanding their distinct impacts on student populations.

Gender-based cultural factors also warrant closer attention. In many contexts, cultural norms influence the underreporting of mental health issues which can distort data and affect the effectiveness of interventions. Future research should examine these gender dynamics to ensure that mental health strategies are inclusive and responsive to all students.

Finally, future studies should prioritize the assessment of detailed interventions, focusing on context, outcomes, and the mechanisms that moderate or mediate their impact. Additionally, investigating the costs and financial sustainability of mental health services is important for ensuring the long-term viability of these support systems.

## 5 Conclusion

The findings from the current study highlight the importance of identifying key personal, cultural, and academic factors that affect students’ well-being, which should inform evidence-based mental health strategies. A comprehensive, inclusive, and multifaceted approach is essential to address the diverse mental health needs of university students, as emphasized in this review. This holistic strategy should involve policy reforms, culturally sensitive interventions, stronger social support systems within universities, and a focus on preventive care and digital innovations. Universities must take a proactive role in fostering environments that promote well-being and offer accessible mental health services. Moreover, international collaboration is crucial for sharing knowledge, developing best practices, improving research methodologies, and addressing global disparities in student mental health care. By implementing these changes, higher education institutions, along with nations, can build a more supportive, accessible, and effective global mental health framework.

## Data Availability

This narrative review synthesizes findings from existing literature, with all sources and data referenced within the article. No new data were generated or analyzed in this study.
